# Dr. Josiah H. Helmer

**Published:** 1904-12

**Authors:** 


Dr. Josiah H. Helmer, a graduate of Albany Medical College (1847), died at Theresa, N. Y., August 20, 1904, aged 83 years. Dr. Helmer was for many years a resident of Lockport, where first he practised his profession and later engaged in banking. His latter years, after retirement from business, were passed at Theresa, where he died.

